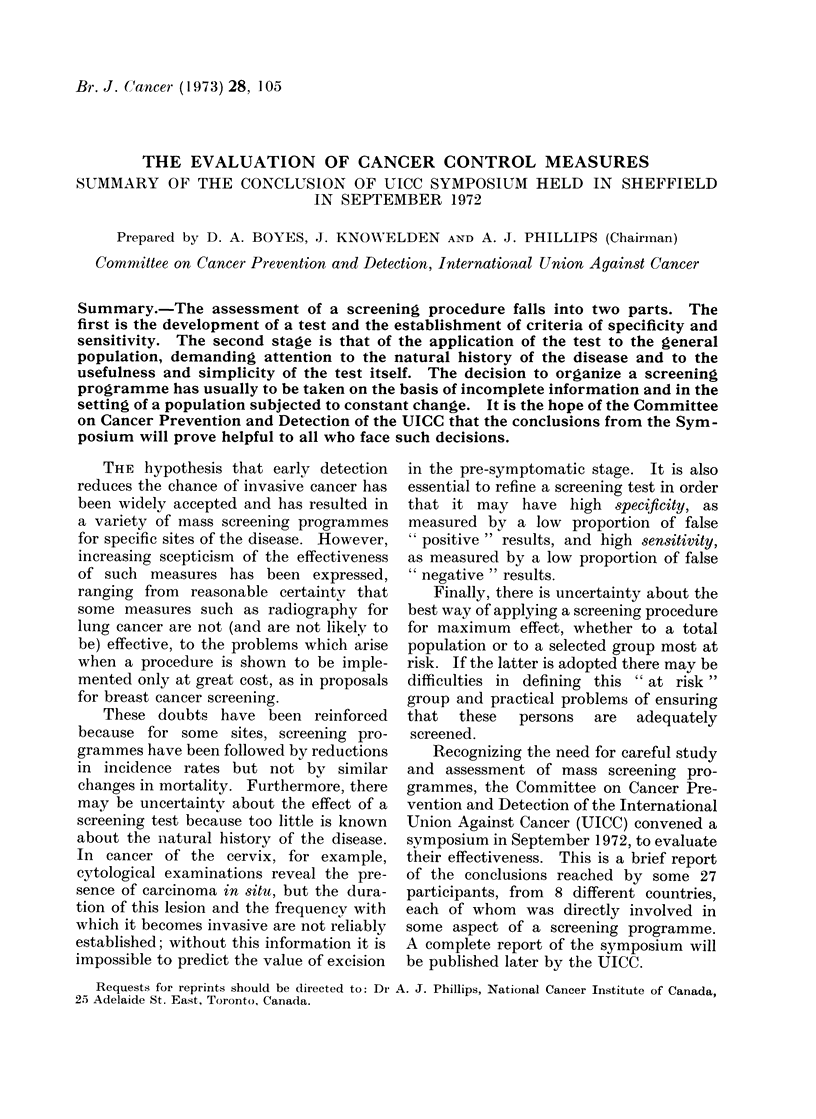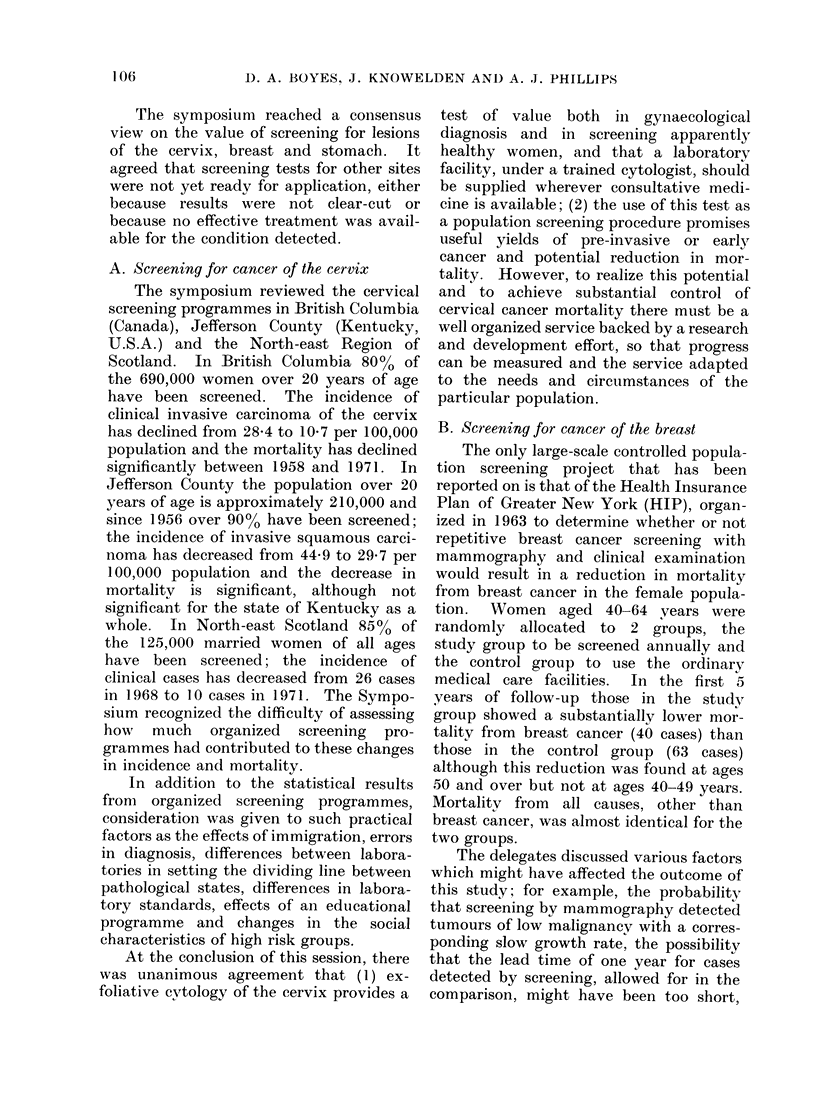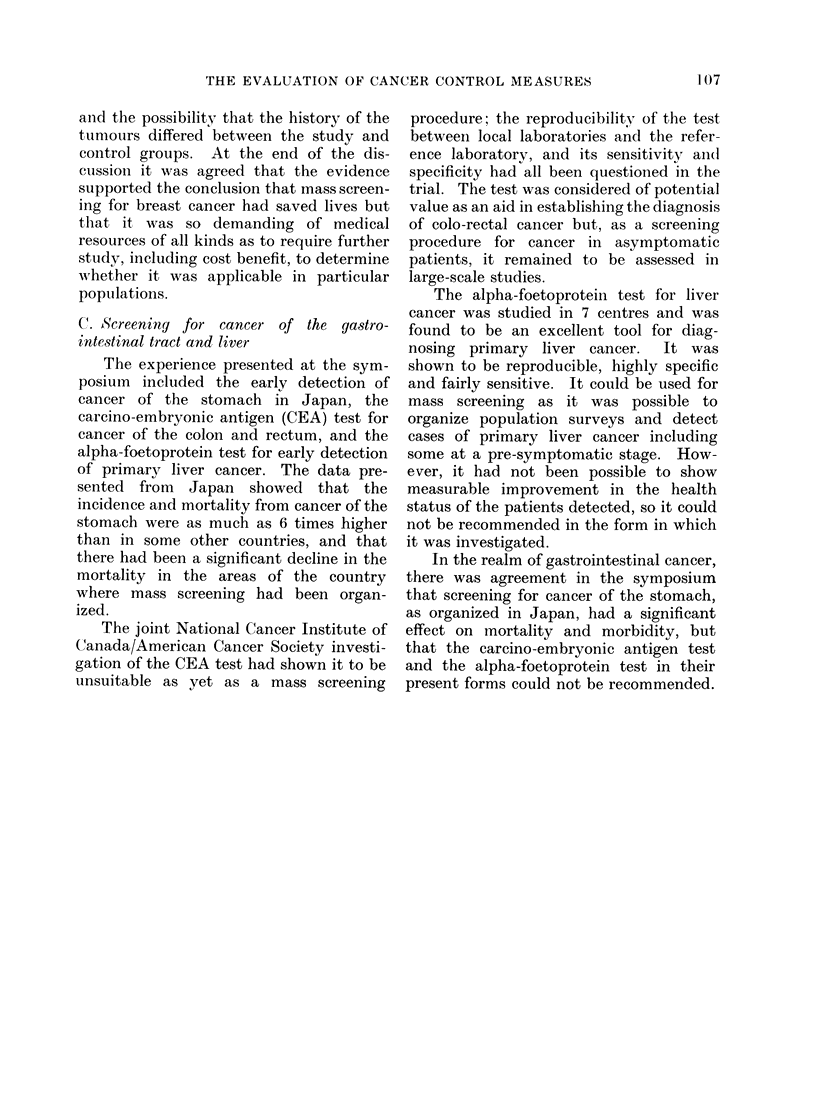# The Evaluation of Cancer Control Measures

**DOI:** 10.1038/bjc.1973.127

**Published:** 1973-08

**Authors:** D. A. Boyes, J. Knowelden, A. J. Phillips

## Abstract

The assessment of a screening procedure falls into two parts. The first is the development of a test and the establishment of criteria of specificity and sensitivity. The second stage is that of the application of the test to the general population, demanding attention to the natural history of the disease and to the usefulness and simplicity of the test itself. The decision to organize a screening programme has usually to be taken on the basis of incomplete information and in the setting of a population subjected to constant change. It is the hope of the Committee on Cancer Prevention and Detection of the UICC that the conclusions from the Symposium will prove helpful to all who face such decisions.


*Br. J. Cancer* (1973) **28**, 105

### THE EVALUATION OF CANCER CONTROL MEASURES

### SUMMARY OF THE CONCLUSION OF UICC SYMPOSIUM HELD IN SHEFFIELD IN SEPTEMBER 1972

Prepared by D. A. BOYES, J. KNOWELDEN AND A. J. PHILLIPS (Chairman)

*Committee on Cancer Prevention and Detection, International Union Against Cancer*

**Summary.—The assessment of a screening procedure falls into two parts. The first is the development of a test and the establishment of criteria of specificity and sensitivity. The second stage is that of the application of the test to the general population, demanding attention to the natural history of the disease and to the usefulness and simplicity of the test itself. The decision to organize a screening programme has usually to be taken on the basis of incomplete information and in the setting of a population subjected to constant change. It is the hope of the Committee on Cancer Prevention and Detection of the UICC that the conclusions from the Symposium will prove helpful to all who face such decisions.**

THE hypothesis that early detection reduces the chance of invasive cancer has been widely accepted and has resulted in a variety of mass screening programmes for specific sites of the disease. However, increasing scepticism of the effectiveness of such measures has been expressed, ranging from reasonable certainty that some measures such as radiography for lung cancer are not (and are not likely to be) effective, to the problems which arise when a procedure is shown to be implemented only at great cost, as in proposals for breast cancer screening.

These doubts have been reinforced because for some sites, screening programmes have been followed by reductions in incidence rates but not by similar changes in mortality. Furthermore, there may be uncertainty about the effect of a screening test because too little is known about the natural history of the disease. In cancer of the cervix, for example, cytological examinations reveal the presence of carcinoma *in situ*, but the duration of this lesion and the frequency with which it becomes invasive are not reliably established; without this information it is impossible to predict the value of excision in the pre-symptomatic stage. It is also essential to refine a screening test in order that it may have high *specificity*, as measured by a low proportion of false " positive " results, and high *sensitivity*, as measured by a low proportion of false " negative " results.

Finally, there is uncertainty about the best way of applying a screening procedure for maximum effect, whether to a total population or to a selected group most at risk. If the latter is adopted there may be difficulties in defining this " at risk " group and practical problems of ensuring that these persons are adequately screened.

Recognizing the need for careful study and assessment of mass screening programmes, the Committee on Cancer Prevention and Detection of the International Union Against Cancer (UICC) convened a symposium in September 1972, to evaluate their effectiveness. This is a brief report of the conclusions reached by some 27 participants, from 8 different countries, each of whom was directly involved in some aspect of a screening programme. A complete report of the symposium will be published later by the UICC.

Requests for reprints should be directed to: Dr A. J. Phillips, National Cancer Institute of Canada, 25 Adelaide St. East, Toronto, Canada.

The symposium reached a consensus view on the value of screening for lesions of the cervix, breast and stomach. It agreed that screening tests for other sites were not yet ready for application, either because results were not clear-cut or because no effective treatment was available for the condition detected.

A. *Screening for cancer of the cervix*

The symposium reviewed the cervical screening programmes in British Columbia (Canada), Jefferson County (Kentucky, U.S.A.) and the North-east Region of Scotland. In British Columbia 80% of the 690,000 women over 20 years of age have been screened. The incidence of clinical invasive carcinoma of the cervix has declined from 28·4 to 10·7 per 100,000 population and the mortality has declined significantly between 1958 and 1971. In Jefferson County the population over 20 years of age is approximately 210,000 and since 1956 over 90% have been screened; the incidence of invasive squamous carcinoma has decreased from 44·9 to 29·7 per 100,000 population and the decrease in mortality is significant, although not significant for the state of Kentucky as a whole. In North-east Scotland 85% of the 125,000 married women of all ages have been screened; the incidence of clinical cases has decreased from 26 cases in 1968 to 10 cases in 1971. The Symposium recognized the difficulty of assessing how much organized screening programmes had contributed to these changes in incidence and mortality.

In addition to the statistical results from organized screening programmes, consideration was given to such practical factors as the effects of immigration, errors in diagnosis, differences between laboratories in setting the dividing line between pathological states, differences in laboratory standards, effects of an educational programme and changes in the social characteristics of high risk groups.

At the conclusion of this session, there was unanimous agreement that (1) exfoliative cytology of the cervix provides a test of value both in gynaecological diagnosis and in screening apparently healthy women, and that a laboratory facility, under a trained cytologist, should be supplied wherever consultative medicine is available; (2) the use of this test as a population screening procedure promises useful yields of pre-invasive or early cancer and potential reduction in mortality. However, to realize this potential and to achieve substantial control of cervical cancer mortality there must be a well organized service backed by a research and development effort, so that progress can be measured and the service adapted to the needs and circumstances of the particular population.

B. *Screening for cancer of the breast*

The only large-scale controlled population screening project that has been reported on is that of the Health Insurance Plan of Greater New York (HIP), organized in 1963 to determine whether or not repetitive breast cancer screening with mammography and clinical examination would result in a reduction in mortality from breast cancer in the female population. Women aged 40–64 years were randomly allocated to 2 groups, the study group to be screened annually and the control group to use the ordinary medical care facilities. In the first 5 years of follow-up those in the study group showed a substantially lower mortality from breast cancer (40 cases) than those in the control group (63 cases) although this reduction was found at ages 50 and over but not at ages 40–49 years. Mortality from all causes, other than breast cancer, was almost identical for the two groups.

The delegates discussed various factors which might have affected the outcome of this study; for example, the probability that screening by mammography detected tumours of low malignancy with a corresponding slow growth rate, the possibility that the lead time of one year for cases detected by screening, allowed for in the comparison, might have been too short, and the possibility that the history of the tumours differed between the study and control groups. At the end of the discussion it was agreed that the evidence supported the conclusion that mass screening for breast cancer had saved lives but that it was so demanding of medical resources of all kinds as to require further study, including cost benefit, to determine whether it was applicable in particular populations.

*C. Screening for cancer of the gastro-intestinal tract and liver*

The experience presented at the symposium included the early detection of cancer of the stomach in Japan, the carcino-embryonic antigen (CEA) test for cancer of the colon and rectum, and the alpha-foetoprotein test for early detection of primary liver cancer. The data presented from Japan showed that the incidence and mortality from cancer of the stomach were as much as 6 times higher than in some other countries, and that there had been a significant decline in the mortality in the areas of the country where mass screening had been organized.

The joint National Cancer Institute of Canada/American Cancer Society investigation of the CEA test had shown it to be unsuitable as yet as a mass screening procedure; the reproducibility of the test between local laboratories and the reference laboratory, and its sensitivity and specificity had all been questioned in the trial. The test was considered of potential value as an aid in establishing the diagnosis of colo-rectal cancer but, as a screening procedure for cancer in asymptomatic patients, it remained to be assessed in large-scale studies.

The alpha-foetoprotein test for liver cancer was studied in 7 centres and was found to be an excellent tool for diagnosing primary liver cancer. It was shown to be reproducible, highly specific and fairly sensitive. It could be used for mass screening as it was possible to organize population surveys and detect cases of primary liver cancer including some at a pre-symptomatic stage. However, it had not been possible to show measurable improvement in the health status of the patients detected, so it could not be recommended in the form in which it was investigated.

In the realm of gastrointestinal cancer, there was agreement in the symposium that screening for cancer of the stomach, as organized in Japan, had a significant effect on mortality and morbidity, but that the carcino-embryonic antigen test and the alpha-foetoprotein test in their present forms could not be recommended.